# Supplementary material for: Origin of Subsequent Malignant Neoplasms in Patients with History of Testicular Germ Cell Tumor
Source: Cancers (Basel). 2020 Dec 14;12(12):3755. doi: 10.3390/cancers12123755 (PMC7764868; doi:10.3390/cancers12123755)
Supplement: Supplementary file 1 [file cancers-12-03755-s001.pdf]

Supplementary Materials

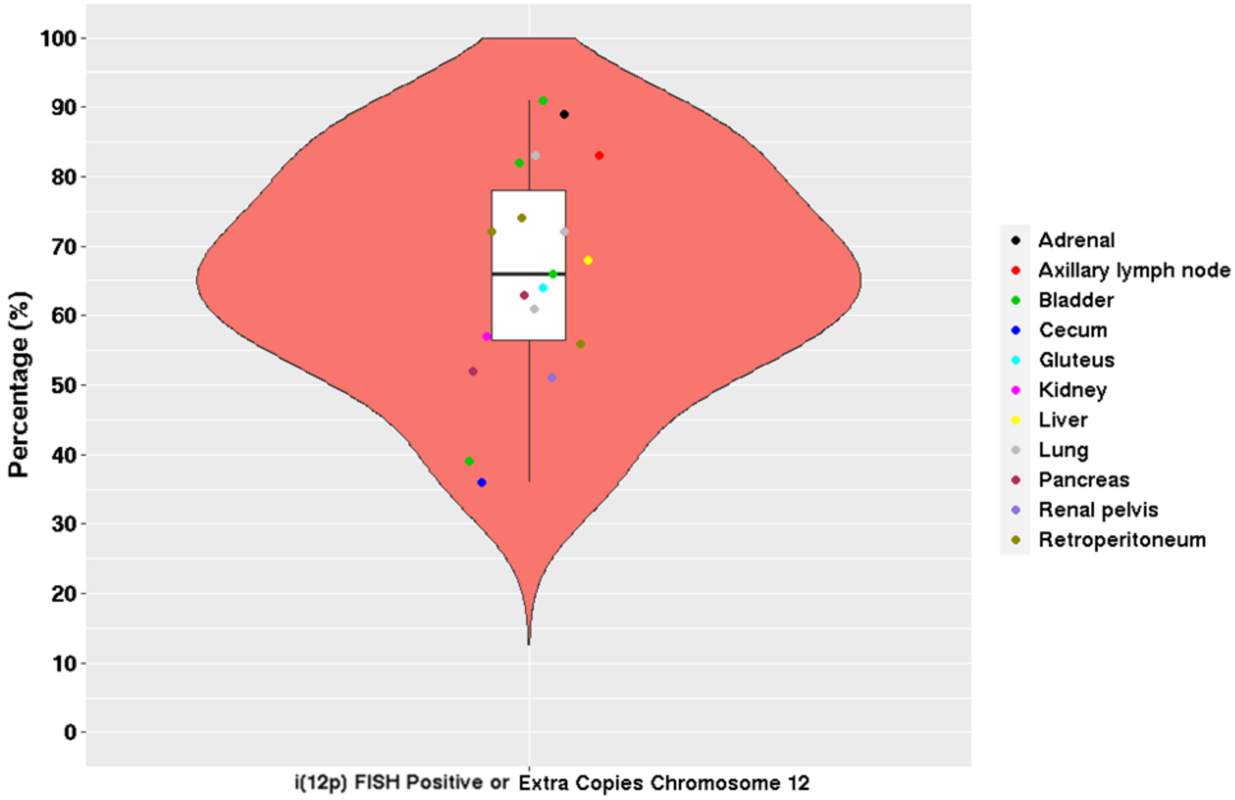

Figure S1a. Violin plot of FISH data plotted by tumor site.

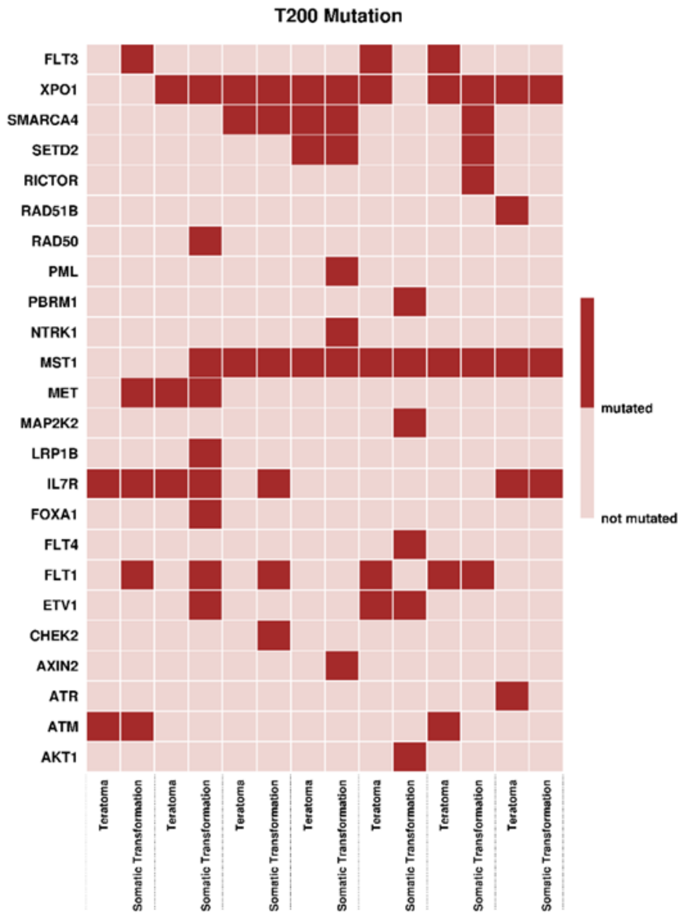

Patient	% Agreement
1	94.8
2	87.9
3	86.2
4	93.1
5	93.1
6	91.4
7	93.1

Figure S1b. Genetic concordance between matched teratoma and somatic transformation specimens. T200 gene panel testing on patient-matched teratoma (n = 7) and somatic transformation tumors (n = 7) demonstrated a high degree of genetic concordance ranging from 86.2% to 94.8%, with no gene consistently mutated across specimens.

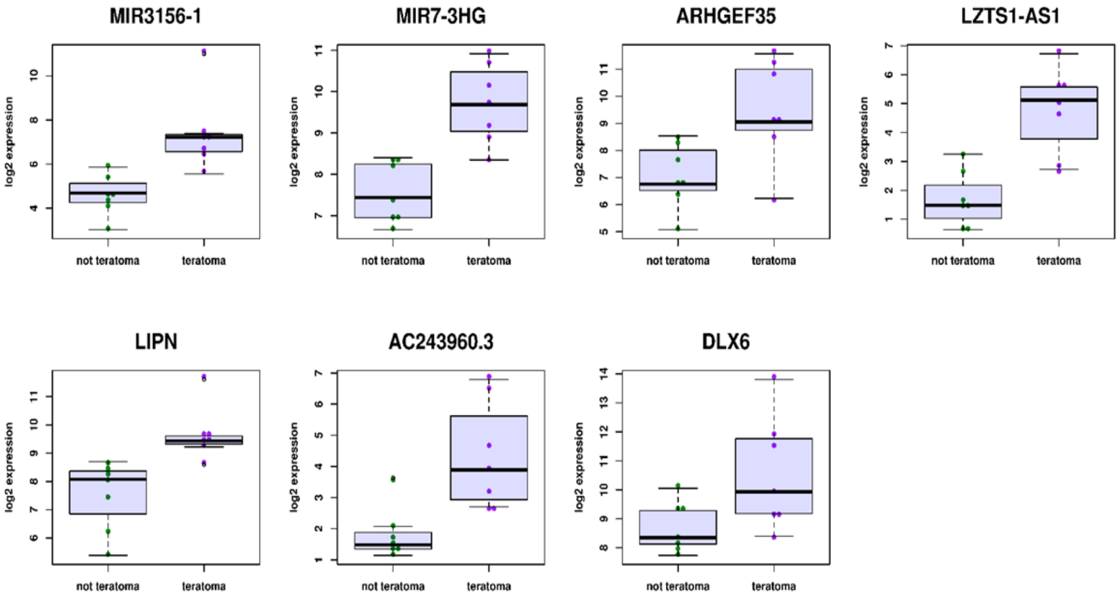

Gene	log2 (Fold Change)	*p*-Value	Adjusted *p*-Value
MIR3156-1	2.04	1.30E-08	0.00057
MIR7-3HG	1.71	2.32E-07	0.00506
ARHGEF35	1.81	4.15E-07	0.00603
LZTS1-AS1	1.94	1.96E-06	0.02134
LIPN	1.59	3.58E-06	0.03128
AC243960.3	1.82	5.74E-06	0.04177
DLX6	1.53	7.38E-06	0.04598

Figure S1c. Differentially expressed genes identified from RNA-Seq.

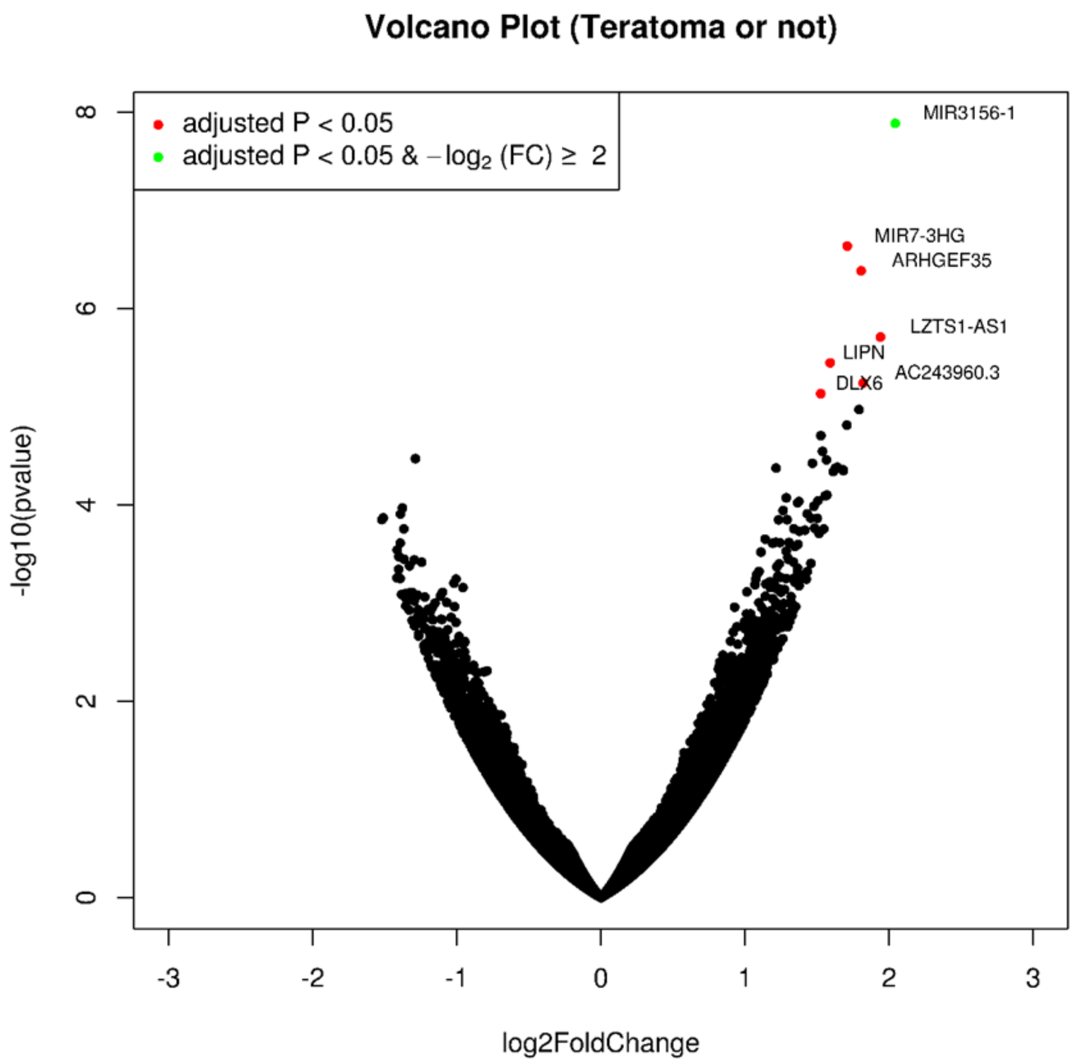

Figure S1d. Volcano Plot: Differential expression of all RNA-Seq genes.

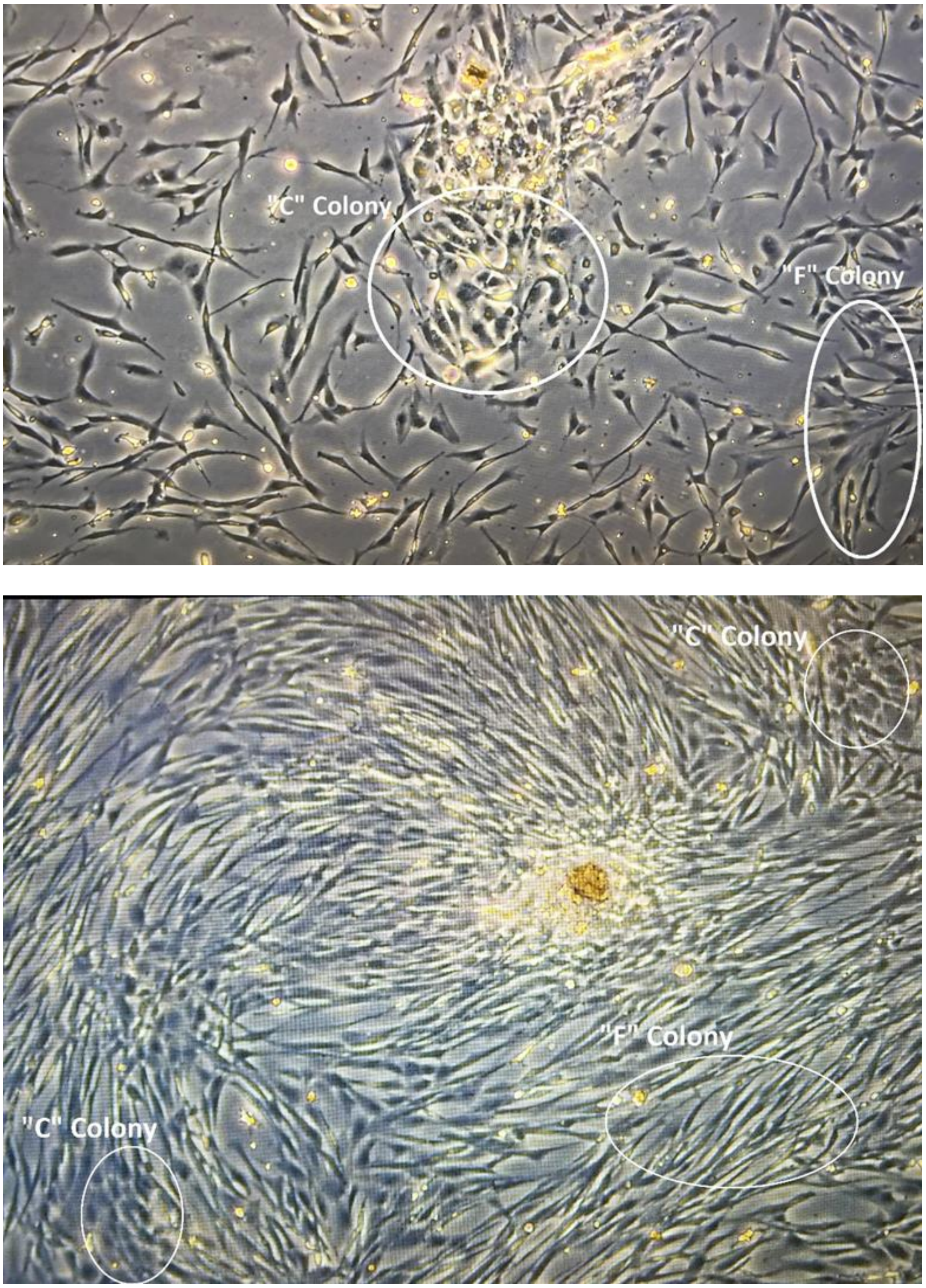

Figure S2. "C" and "F" cell colonies forming in DMEM base media. (**a**) Representative image of Tera13 growing in DMEM + glutamate + non-essential amino acids at passage 0, day 6. (**b**) Representative image of Tera13 growing in DMEM + glutamate + non-essential amino acids at passage 0, day 9.

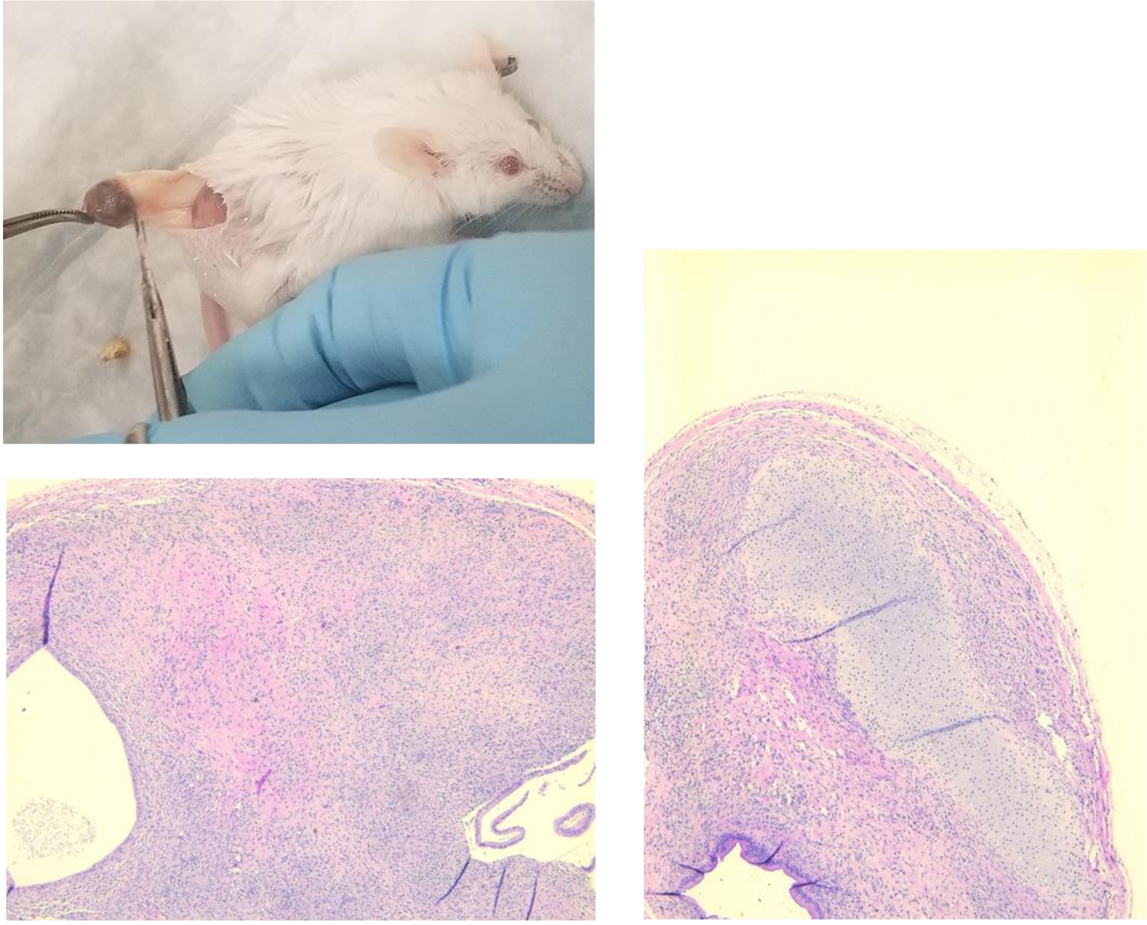

Figure S3. Resection of tumor from SCID mouse and representative H&E slides of the tumor after xenograft injection with Tera13. All three injected mice had microscopic evidence of teratoma.

(a)

Cell Line	NANOG	OCT 3/4	SALL4
Tera13	+	-	-
Tera22	-	-	-

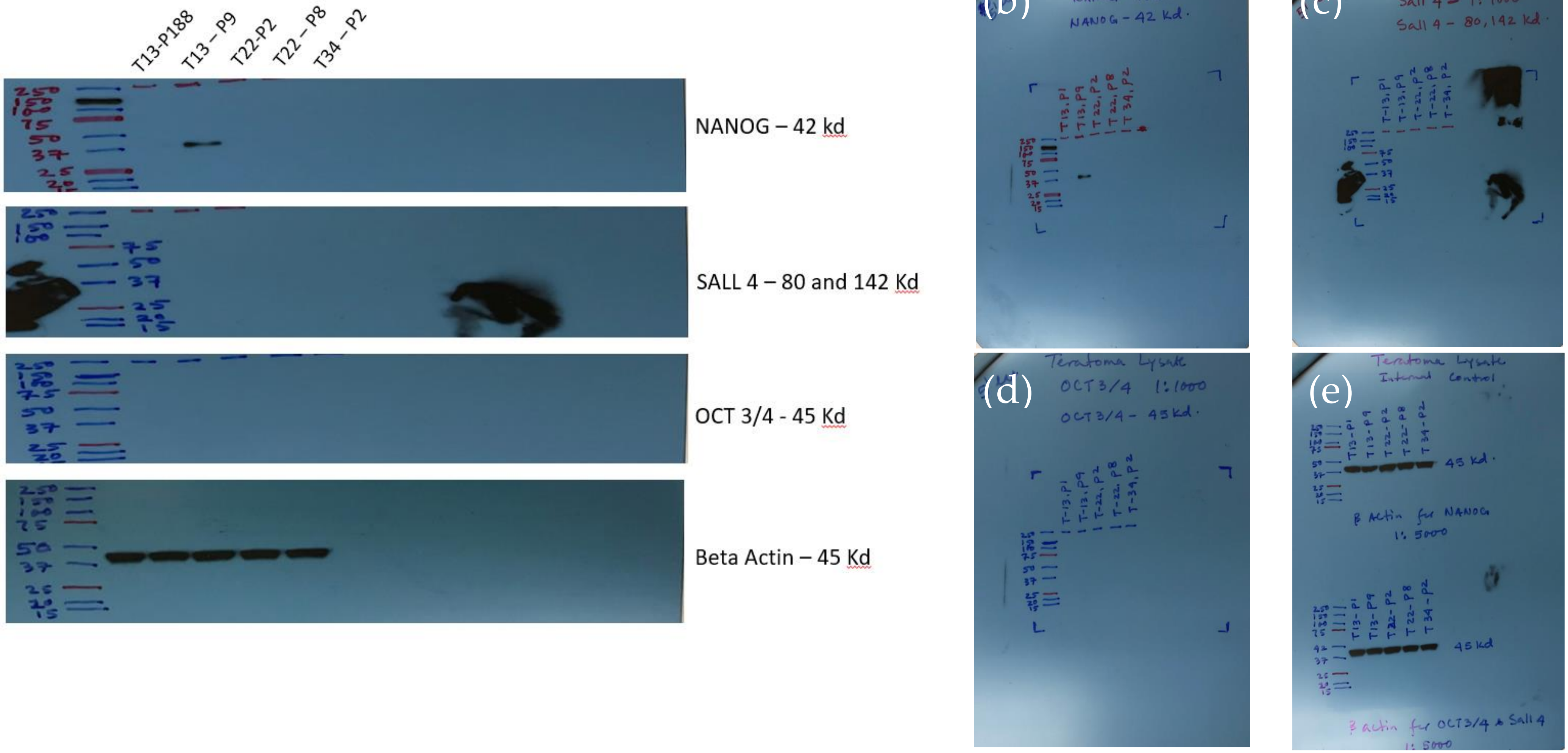

Figure S4. Western blots. (**a**) Summary of Western blots from teratoma cell lines. Detected NANOG but not OCT3/4 or SALL4 in Tera13, and did not detect NANOG, OCT3/4, or SALL4 in Tera22. Molecular weight ladders provided on left. (**b**) Uncropped Western blots for NANOG. (**c**) Uncropped Western blots for SALL4. (**d**) Uncropped Western blots for OCT3/4. (**e**) Uncropped Western blots for teratoma lysate (internal control) and Beta Actin.

Table S1. Clinical characteristics and chemotherapy regimens of matched teratoma and somatic transformation specimens.

Case No.	Age	Primary	Stage	Metastatic Site	Chemotherapy	Somatic Histology	Survival(mo.)
21	21	ST	IIIC	Retroperitoneum	BEP x4, VIP x4	Adenocarcinoma	17
22	42	ST	IIIB	Retroperitoneum	BEP/EP, TIP, CISCA, ACE, ATP, IAG	Carcinoma/sarcoma	18
23	23	ES	IB	Retroperitoneum	VIP, BEP x4, TIP x2, IAG	PNET	33
24	20	EYST	IIIB	Retroperitoneum	BEP x4, TIP x2, VeIP x2, ACE + thalidomide, paclitaxel/gemcitabine + bevacizumab	Sarcoma	24
25	27	ECYT	IIIB	Lung	BEP/EP x4	Adenocarcinoma	53+
26	24	EYST	IIIA	Mediastinum	BEP/EP x4	Rhabdomyosarcoma	23+
27	27	EYST	IIIC	Retroperitoneum	BEP x4, ATP x2, ACE x6, CISCA x1	Angiosarcoma	19+

Age and clinical stage were at time of diagnosis; survival was from time of diagnosis. T, teratoma; PNET, primitive neuroectodermal tumor; ST, seminoma teratoma; ES, embryonal carcinoma, seminoma; EYST, embryonal carcinoma, yolk sac tumor, seminoma, teratoma; ECYT, embryonal carcinoma, choriocarcinoma, yolk sac tumor, teratoma. BEP/EP, bleomycin, etoposide, cisplatin/etoposide/cisplatin; VIP, etoposide, ifosphamide, cisplatin; TIP, paclitaxel, ifosphamide, cisplatin; CISCA, cyclophosphamide, doxorubicin, cisplatin; ACE, actinomycin D, cyclophosphamide, etoposide; ATP, doxorubicin, paclitaxel, cisplatin; IAG, ifosphamide, doxorubicin, gemcitabine; VeIP, vinblastine, ifosphamide, cisplatin.

Table S2. Clinical characteristics of patients from whom cell lines Tera13 and Tera21 derived.

	**Patient 28 (Tera13)**	**Patient 29 (Tera21)**
Age at diagnosis (years)	27	22
Initial stage	IA	IIIB
TGCT subtype	NSGCT	NSGCT
Primary<br>Size (cm)<br>Pathology	4.6<br>Embryonal carcinoma 30%<br>Yolk sac tumor 10%<br>Teratoma 60%	NA*
Tumor markers<br>Beta HCG (mIU/mL)<br>AFP (ng/mL)<br>LDH (U/L)	S1<br>NL<br>NL<br>NL	S2<br>2,616<br>2,712<br>1,279
Initial therapy	Surveillance	BEP x4
Time to recurrence (mos.)	12	
Recurrence stage	IIIB	
Tumor markers<br>Beta HCG (mIU/mL)<br>AFP (ng/mL)<br>LDH (U/L)	S2<br>963<br>3,603<br>NA	
Recurrence therapy	EP x4	
Post-chemotherapy<br>Site<br>Size (cm)<br>Pathology	 Retroperitoneum<br>9.0<br>Teratoma	 Pelvis<br>4.5<br>Teratoma

TGCT, germ cell tumor of the testis; NSGCT, non-seminomatous germ cell tumor; BEP, bleomycin, etoposide, cisplatin; EP, etoposide, cisplatin; NL, within normal limits; NA, not available. *- Microlithiasis in left testis, suspect burnt-out tumor.

Supplementary Methods

FISH MDACC Probe Cutoffs: The normal cut off for i(12p) probe established at 95% ($p < 0.05$) confidence level was 15.5% for three 12p13.3 and two D12Z3 signals; 12.4% for two 12p13.3 and one D12Z3 signals; 10% ≥4 12p13.3 and two D12Z3 signals; 8.3% for one 12p13.3 and two D12Z3 signals; 12.9% for monosomy of 12p13.3/12cen; and 15.8% for polyploidy of 12p13.3/12cen probe.

RNA and cDNA Library Preparation and Capture. A mapped H&E-stained slide and unstained sections of the teratoma and its associated somatic transformation in the same tumor were obtained from paraffin blocks. After isolation, RNA was quantified by PicoGreen (Invitrogen) and quality was assessed using the 2200 TapeStation (Agilent). RNA from each sample (10–100 ng) was converted into double-stranded cDNA using Ovation RNA-Seq System V2 kit from Nugen. cDNA was sheared by sonication. The sheared DNA proceeded to library prep using KAPA library prep hyper kit (KAPA) following the "with beads" manufacturer protocol. Samples were analyzed on TapeStation to verify correct fragment size and to ensure the absence of extra bands. Samples were quantified using KAPA qPCR quantification kit. Equimolar amounts of DNA were pooled for capture (2–6 samples per pool). We used whole-exome biotin-labeled probes from Roche NimbleGen (Exome V3) and followed the manufacturer's protocol for the capture step. The quality of each captured sample was analyzed on TapeStation using the High Sensitivity DNA kit and the enrichment was accessed by qPCR using specific primers designed by Roche NimbleGen. The cutoff for the enrichment was 50 fold minimum.

DNA/RNA Sequencing Analysis and Interpretation. For DNA analysis, the T200 target-capture deep-sequencing data were aligned to human reference assembly hg19 using BWA, and duplicated reads were removed using Picard [1,2]. Single nucleotide variants (SNVs) and small indels were

identified using an in-house-developed analysis pipeline which classified variants into somatic, germline, and loss of heterozygosity based on variant allele frequencies in the tumor and the matched normal tissues. Copy number alterations were determined using a previously published algorithm which reported gain or loss status of each exon [3]. Potential functional consequence of detected variants were determined by comparing them with those in dbSNP, COSMIC, [4] and TCGA databases, and annotating them using the VEP [5] and other programs. For RNA analysis, we used a bioconductor package DESeq2 (with adjusted p-value < 0.05) to identify differentially expressed genes [6]. We used VirusFinder [7] (version 2.0) to align reads that did not map to the human reference genome [8].

Post-Chemotherapy Teratoma Cell Line Development and Evaluation

Teratoma Specimens. Post-chemotherapy residual masses were surgically resected and tissue collected from patients undergoing post-chemotherapy retroperitoneal lymphadenectomy or mass excision. The cohort contained 14 patients, of which 6 were confirmed teratoma on pathology and 5 demonstrated fibrosis/necrosis. Three patients had viable tumor or malignant transformation and were not included in cell culture creation. Using the techniques described below, teratoma-derived cells were successfully propagated from 2/6 of these teratoma cases, which were labelled Tera13 and Tera22. These were studied in more detail by flow cytometry, as described below. The Tera13 cell culture was further examined by orthotropic xenografting primary and from in-vitro culture as described below and in the manuscript.

Cell Culture. After obtaining fresh tissue sample and transporting it to the lab in PBS solution, nodular areas of visually presumed teratoma tissue were aseptically cut into 1–2 mm pieces. This occurred within 90 min of surgery and was then washed with 2–3 times volume PBS and 200 μg penicillin/streptomycin. The minced sample was digested for 30–180 minutes in a 50 mL conical tube with PBS+caseinase+DNAse (Sigma-Aldrich) at a concentration of 1 mL of each enzyme for 50 mL PBS. Incubation at 37 °C in 10% CO_2 was completed to dissociate the sample with periodic gentle shaking and checking tissue breakdown. After adequate dissociation, the suspension was centrifuged at 2000 RPM for 2 min to pellet the cells. The supernatant was removed and washed once with PBS. Culture media was added to cell pellet, mixed, and transferred to a laminin-coated T75 flask (Sigma-Aldrich, St. Louis, U.S.A.) for growth. In total, 7 mL of cells/media was added to each T75. Cultures were incubated at 37 °C in 10% CO_2, 5% O_2, and medium was changed every second day. Cultures were successful in DMEM (Gibco, Waltham, U.S.A.) and STEMium (ScienCell, Carlsbad, U.S.A.) bases with the addition of 40 nM L-glutamine and penicillin/streptomycin. In DMEM base, cultures grew “C” colonies and “F” colonies within 1 week; however, in STEMium base, only “C” colonies formed, no matter the length of time. At 90% confluence, DMEM based cultures were passed and split into multiple T75 flasks. In STEMium, colonies grew very slowly and some were changed to DMEM base, at which point “F” colonies began to propagate.

Flow Cytometry. Cell colonies from Tera13 and Tera22 were cultured in DMEM media until ~80% confluent. Samples were then prepared with antibody dilutions per the manufacturer’s recommendations. Live cell cultures were examined by flow cytometry unstained and with DAPI for control. Then, DAPI was added to each antibody, as they were examined independently and together.

Xenograft. Secondary PDX models were created with Tera13 cells. Cell culture was suspended in DMEM and incubated overnight in Matrigel growth media. SCID male mice were anesthetized with ketamine/medetomidine and prepped for sterility according to approved protocols. A 1–2 mm incision was made in the midline lower back and subdermal injection of cell with Matrigel was injected in the bilateral flanks. Flank mass measurements were completed every 2 days once palpable, which was approximately 4–6 weeks after the initial injection.

There were no control groups of mice undergoing sham PDX modeling, as this was unnecessary for this study. Three mice were kept together as an experimental unit in a single cage. The number of mice was determined based on previous experience with PDX modeling and not detailed out a priori. During experimental design we decided to include young, male SCID mice secondary to testicular cancer and available mice in our existing laboratory with immunodeficiency. Data point collection

included tumor size with measurements every 2–3 days and veterinarian assessment of overall mouse vitality. Mice were monitored for distress or burden from tumor size over time. If at any time the mice reveal evidence of behavior changes suggestive of suffering, they were euthanized via the standard MD Anderson protocol for the fair treatment of animals.

Ethics Statement. All human samples were anonymously coded and obtained according to MDA IRB# LAB02-0152. Ethical use of immune-deficient mice for the transplantation of human teratoma cells was authorized by MD Anderson.

References

1. Depristo, M.A.; Banks, E.; Poplin, R.; Garimella, K.V.; Maguire, J.R.; Hartl, C.; Philippakis, A.A.; Del Angel, G.; Rivas, M.A.; Hanna, M.; et al. A framework for variation discovery and genotyping using next-generation DNA sequencing data. *Nat. Genet.* **2011**, *43*, 491–498, doi:10.1038/ng.806.
2. Li, H.; Handsaker, B.; Wysoker, A.; Fennell, T.; Ruan, J.; Homer, N.; Marth, G.; Abecasis, G.; Durbin, R; 1000 Genome Project Data Processing Subgroup. The Sequence Alignment/Map format and SAM tools. *Bioinformatics* **2009**, *25*, 2078–2079, doi:10.1093/bioinformatics/btp352.
3. Lonigro, R.J.; Grasso, C.S.; Robinson, D.R.; Jing, X.; Wu, Y.-M.; Cao, X.; Quist, M.J.; Tomlins, S.A.; Pienta, K.J.; Chinnaiyan, A.M. Detection of somatic copy number alterations in cancer using targeted exome capture sequencing. *Neoplasia* **2011**, *13*, 1019–IN21, doi:10.1593/neo.111252.
4. Bamford, S.; Dawson, E.; Forbes, S.; Clements, J.; Pettett, R.; Dogan, A.; Flanagan, A.; Teague, J.; Futreal, P.A.; Stratton, M.R.; et al. The COSMIC (Catalogue of Somatic Mutations in Cancer) database and website. *Br. J. Cancer* **2004**, *91*, 355–358, doi:10.1038/sj.bjc.6601894.
5. McLaren, W.; Pritchard, B.; Rios, D.; Chen, Y.; Flicek, P.; Cunningham, F. Deriving the consequences of genomic variants with the Ensembl API and SNP Effect Predictor. *Bioinformatics* **2010**, *26*, 2069–2070, doi:10.1093/bioinformatics/btq330.
6. Love, M.I.; Huber, W.; Anders, S. Moderated estimation of fold change and dispersion for RNA-seq data with DESeq2. *Genome Biol.* **2014**, *15*, 550.
7. Wang, Q.; Jia, P.; Zhao, Z. VirusFinder: Software for efficient and accurate detection of viruses and their integration sites in host genomes through next generation sequencing data. *PLoS ONE* **2013**, *8*, e64465, doi:10.1371/journal.pone.0064465.
8. Bhaduri, A.; Qu, K.; Lee, C.S.; Ungewickell, A.; Khavari, P.A. Rapid identification of non-human sequences in high-throughput sequencing datasets. *Bioinformatics* **2012**, *28*, 1174–1175, doi:10.1093/bioinformatics/bts100.
